# Comparative Study of Polymer of Intrinsic Microporosity-Derivative Polymers in Pervaporation and Water Vapor Permeance Applications

**DOI:** 10.3390/polym16202932

**Published:** 2024-10-18

**Authors:** Esra Caliskan, Sergey Shishatskiy, Volkan Filiz

**Affiliations:** Institute of Membrane Research, Helmholtz-Zentrum Hereon, Max-Planck-Str. 1, 21502 Geesthacht, Germany; esra.caliskan@hereon.de (E.C.); sergey.shishatskiy@hereon.de (S.S.)

**Keywords:** polymers of intrinsic microporosity (PIM-1), membranes, gas and vapor permeance, pervaporation

## Abstract

This study assesses the gas and water vapor permeance of PIM-derivative thin-film composite (TFC) membranes using pervaporation and “pressure increase” methods, and provides a comparative view of “time lag” measurements of thick films obtained from our previous work. In this study, TFC membranes were prepared using PIM-1 and homopolymers that were modified with different side groups to explore their effects on gas and water vapor transport. Rigid and bulky aliphatic groups were used to increase the polymer’s free volume and were evaluated for their impact on both gas and water transport. Aromatic side groups were specifically employed to assess water affinity. The permeance of CO_2_, H_2_, CH_4_ and water vapor through these membranes was analyzed using the ‘pressure increase’ method to determine the modifications’ influence on transport efficiency and interaction with water molecules. Over a 20 h period, the aging and the permeance of the TFC membranes were analyzed using this method. In parallel, pervaporation experiments were conducted on samples taken independently from the same membrane roll to assess water flux, with particular attention paid to the liquid form on the feed side. The significantly higher water vapor transport rates observed in pervaporation experiments compared to those using the “pressure increase” method underline the efficiency of pervaporation. This efficiency suggests that membranes designed for pervaporation can serve as effective alternatives to conventional porous membranes used in distillation applications. Additionally, incorporating “time lag” results from a pioneering study into the comparison revealed that the trends observed in “time lag” and pervaporation results exhibited similar trends, whereas “pressure increase” data showed a different development. This discrepancy is attributed to the state of the polymer, which varies significantly depending on the operating conditions.

## 1. Introduction

One of the most important issues of this century is access to clean water. Factors such as climate change, global population growth, and industrialization issues are increasingly straining water resources, while freshwater reserves and traditional energy sources are rapidly depleting. It is therefore crucial to develop energy-efficient water treatment technologies that are able to utilize energy from renewable sources such as wind and solar light, thus reducing environmental impacts [1,2,3]. Membrane technology, recognized for its high energy efficiency and low carbon footprint, has garnered significant attention for water treatment applications. Its advantages include easy operability and low cost when compared to traditional thermal processes. Membranes are widely used in all technological stages of the water treatment process starting from micro- and ultrafiltration and up to nanofiltration and reverse osmosis. The last two processes give high-quality water but are energy-intensive [4,5,6]. The relatively new process of membrane distillation offers high-quality water production and can utilize lower-quality water sources compared to methods like reverse osmosis. Water desalination is one of the areas where membrane distillation has been used effectively. In recent applications in desalination, it has proven to be particularly effective in producing high-quality fresh water from brackish sources using waste heat from adjacent industrial processes [7,8]. For example, several desalination plants in Middle East regions rich in solar energy resources have integrated solar-powered membrane distillation systems to maximize the use of renewable waste heat [9]. Additionally, desalination requires a lower grade of energy, even allowing for the use of waste heat, to carry out the separation process. It is usual practice to use porous membranes for this process, which requires one to face certain drawbacks such as, e.g., fouling of the porous structure with organic or inorganic matter present in the feed water causing water breakthrough through the membrane and compromising the product. It is considered favorable to develop a new type of membrane with a highly permeable, hydrophobic, defect-free selective layer, which would provide high water flux through the membrane and would not allow any impurities of biological or inorganic nature to move through it [10,11,12].

One of the widely studied polymer types are polymers of intrinsic microporosity (PIMs). These polymers have received considerable attention in recent years due to their very high free volume, making them desirable for gas separation applications [13]. Despite the primary application of PIM-1 in dense films for gas separation due to its intrinsic microporosity and solvent resistance, its unusual properties have led researchers to explore different membrane preparations and applications, such as pervaporation for toluene recovery [14] or alcohol–water separation [15,16]. In addition to pervaporation, previous studies have explored various modifications of PIM-1 for nanofiltration. One study enhanced water flow by carbonizing PIM-1, while another developed carboxylate-functionalized PIM-1 for similar purposes [17,18].

In our previous study, the hydrophobic and high-free-volume PIM-1 polymer was examined for the possibility to fabricate a porous membrane via the phase-inversion process and its suitability in membrane distillation (MD), which was shown to be promising [19]. Additionally, a thin-film composite (TFC) membrane was formed by deposition of PIM-1 selective layer formation on a PAN ultrafiltration membrane as a support, and its water permeance was compared with that of the porous PIM-1 membrane. While the porous membrane exhibited a higher water vapor permeance of 100 m^3^(STP) m^−2^ h^−1^ bar^−1^, the TFC membrane displayed a considerable vapor permeance of 35 m^3^(STP) m^−2^ h^−1^ bar^−1^. Additionally, in another study [20], our research group utilized a set of anthracene maleimide monomers with aliphatic side groups of varying sizes and shapes as precursors for a series of polymer of intrinsic microporosity (PIM)-based homopolymers. These side groups, which did not exceed 7% of the monomer’s molecular weight, were investigated for their effect on the gas transport properties of homopolymers. It was observed that particularly the bulky side groups significantly enhanced the gas transport. Accordingly, the permeability coefficients for CO_2_ and CH_4_ in isotropic films made from the homopolymer derived from the *t*-butyl substituted comonomer (*t*-butyl-100) were the highest among the studied homopolymers.

Building upon aforementioned research, the current study investigates the formation of TFC membranes with a selective layer made of anthracene maleimide-modified PIM homopolymers to examine how polymer gas and water vapor transport properties are retained or altered upon transformation into thin layers, and how these properties change with time. Characterized by minimal polymer usage per unity of membrane surface due to the formation of exceptionally thin selective layers, TFC membranes are widely adopted in industrial gas and water vapor separation applications. This paper utilizes the “pressure increase” method to determine water vapor, carbon dioxide, hydrogen, and methane permeances, allowing for a detailed comparison of the permeation properties of TFC membranes and thick isotropic films reported earlier. Additionally, this study evaluates how TFC membrane permeance evolves over time in relation to the nature of the penetrant and of the chemical composition of the polymer of the selective layer. Therefore, the research aims to explore how the side groups of these polymers affect membranes’ physical aging and overall performance in applications such as pervaporation and general water vapor permeation. By comparing the properties of thin-film composite membranes to those of previously studied PIM membranes, this paper seeks to determine whether modifications in side groups can enhance membrane performance and mitigate the challenges posed by physical aging.

The analysis is enriched by including data from pervaporation experiments, providing a framework to assess vapor permeation data from both pervaporation and “pressure increase” methods. The study addresses challenges in data comparison between these two methods due to differences in measurement units. Moreover, the use of saturated vapor pressure in pervaporation experiments suggests significantly higher permeance values compared to those from “pressure increase” experiments, highlighting the need for an understanding of membrane behavior at the interface where water transitions from a liquid to a vapor state [21]. This dynamic is critical as it implies a potential disparity in how membranes behave under different operational conditions and measurement setups [22]. Overall, this study explores how changes at the molecular level affect the macroscopic properties of membranes, particularly their longevity and efficiency in separation processes. The outcomes could lead to an improved membrane separation process for water treatment technologies that is better suited to meet the demands of modern industrial applications.

## 2. Materials and Methods

### 2.1. Materials

5,5′,6,6′-tetrahydroxy-3,3,3′,3′-tetramethyl-1,1′-spirobisindane (TTSBI, 98%) was purchased from ABCR GmbH (Karlsruhe, Germany). 2,3,5,6-tetrafluoro-terephthalonitrile (TFTPN, 99%) was obtained from Lanxess (Cologne, Germany). TFTPN was sublimated twice at 70 °C under vacuum before use. Potassium carbonate (K_2_CO_3_, 99%) and dimethyl acetamide (DMAc, 99%) were purchased from Alfa Aesar (Karlsruhe, Germany). All other commercially available reagents were obtained from Merck (Darmstadt, Germany) and were used without further treatment.

### 2.2. Synthesis of Polymers

#### 2.2.1. PIM-1 Synthesis

PIM-1 was produced utilizing a rapid synthesis technique as previously described [20,23]. A mixture of TTSBI (20 mmol) and TFTPN (20 mmol) was prepared in a three-necked round bottom flask, dissolved in 65 mL of dimethylacetamide (DMAc) under an argon atmosphere. The addition of K_2_CO_3_ (44 mmol) changed the color of the reaction from orange to yellow. The flask was then quickly placed in an oil bath preheated to 150 °C. To prevent PIM-1 precipitation and enhance the formation of a higher-molecular-weight polymer, diethylbenzene (DEB) (15 mL) was added gradually. After stirring the mixture for 30 min, the reaction was stopped by precipitating the mixture in methanol. The precipitated solid was filtered and dried at 80 °C under vacuum. This dried polymer was then dissolved in CHCl_3_, re-precipitated in methanol, collected by filtration, and dried at 80 °C under high vacuum to yield the final yellow solid product with a 90% yield. The molecular weight (Mw) and dispersity (*Ð*) of the purified polymer were determined using size-exclusion chromatography, resulting in an Mw of 132.5 kg/mol and a *Ð* of 4.9.

#### 2.2.2. Synthesis of Homopolymers

**Figure 1:**
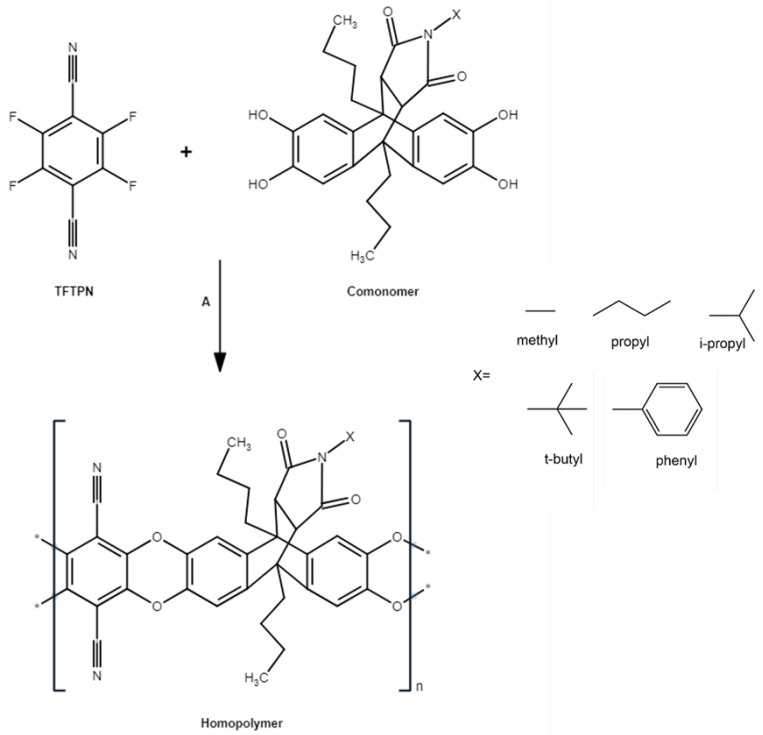
Synthesis of homopolymer. A: reagents and conditions DMAc, K_2_CO_3_, 150 °C, 30–60 min.

The synthesis and characterization of anthracene maleimide-structured bulky and linear group monomers, as well as the homopolymers derived from these monomers, were reported in our previous study [20]. Accordingly, the homopolymer series was synthesized through the polycondensation of comonomers, which are essentially TTSBI substitutions, with TFTPN using a method akin to that employed for PIM-1 synthesis. These comonomers were specifically modified by incorporating an anthracene maleimide structure with various aliphatic side groups. The detailed synthesis scheme is provided in Figure 1. The nomenclature for homopolymers was adapted from the reference study. In order to clarify the nomenclature of the homopolymers used in this study, they were identified as methyl-/propyl-/*i*-propyl-/*t*-butyl-/phenyl-100. The suffix “-100” represents the comonomer indicating that, for example, methyl-100 comprises 100 mol% comonomer unit.

### 2.3. Preparation of Thin-Film Composite (TFC) Membranes

The fabrication of the TFC membrane was carried out using a widely adopted dip-coating technique, the most used for TFC membrane formation [24]. This method entailed placing the porous substrate in contact with the polymer solution, then uniformly withdrawing it from the solution at a predetermined speed to ensure an even coating of the polymer solution on the ultrafiltration (UF) membrane used as a support for a selective layer. In our experiment, a dense selective layer of PIM-1 or homopolymer was applied over a porous polyacrylonitrile (PAN) UF membrane using an in-house-designed laboratory scale membrane casting machine. A 1% wt. solution of polymer in THF was prepared, which upon filtration was poured into a uniquely designed bath. Subsequently, the PAN membrane was aligned with this solution. A consistent meniscus of the polymer solution was formed between the solution surface and PAN membrane by slightly lowering the bath by about 2 mm. By drawing the porous substrate out of the meniscus at a fixed pace, a thin film of the solution was formed on the support’s surface. The drying of the TFC membrane was conducted at ambient conditions, letting the solvent evaporate freely without any active control over the process. This step was performed in an enclosure with substantial low-humidity air flow to prevent the accumulation of solvent vapors around the casting device, aside from the immediate area of the polymer solution bath. The prepared membrane was dried for 1 h before gas transport experiments by the “pressure increase” or pervaporation methods were conducted.

### 2.4. Characterization Methods

#### 2.4.1. Size Exclusion Chromatography (SEC)

Size exclusion chromatography (SEC) analyses were conducted at 30 °C using chloroform (CHCl_3_) as the solvent. The chromatographic setup included a series of columns: a precolumn-SDV-linear, an SDV-linear, and an SDV with a 102 nm pore size, each having an inner diameter of 4.6 mm and a length of 53 cm, supplied by Polymer Standard Service GmbH, Mainz, Germany. The flow rate was maintained at 1.0 mL per minute. To detect the concentration of polymers, both refractive index (RI) and ultraviolet (UV) detectors were utilized. Calibration with polystyrene standards allowed for the determination of the apparent weight average molecular weight (Mw) and the dispersity index (*Ð*) of the synthesized polymers.

#### 2.4.2. Gas and Water Vapor Permeance Measurements: “Pressure Increase” Method

Gas transport properties of thin-film composite (TFC) membranes were measured using a “pressure increase” method, which follows the “constant volume/variable pressure” principle as described elsewhere [25,26]. This technique is especially suitable for membranes with thin selective layers and allows for the investigation of membrane permeances in a wide range of feed pressures and temperatures. Additionally, this method was utilized for water vapor transport studies in TFC membranes under controlled conditions (40 °C, feed vapor pressure below 60 kPa, permeate pressure 1–13 mbar), and the permeance from at least ten measurements were averaged One limitation of this method is that it cannot assess vapor permeance in saturated vapor conditions because the setup isolates the gas or vapor in the feed vessel from any external supply, leading to a slight pressure drop at the start. This pressure drop prevents the execution of experiments with vapors at activities above 90%. However, isolating the feed volume from the supply line allows experiments to be conducted over a wide range of vapor activities, decreasing from high to low. The lowest feed vapor activity to be investigated is determined by the initial conditions of the experiment. The program conducting the experiment is set to measure the permeance value by collecting penetrant on the permeate side of the setup, within a range of 1 to 10 mbar. This final value determines the lowest feed pressure, as the program is designed to stop the experiment when the feed pressure equals the desired permeate pressure. However, this feature provides vital insights into membrane performance as feed pressure decreases, which is essential for studying substances that are highly soluble or prone to condensation [19,27].

#### 2.4.3. Water Vapor Permeance Measurement: Pervaporation Method

**Figure 2:**
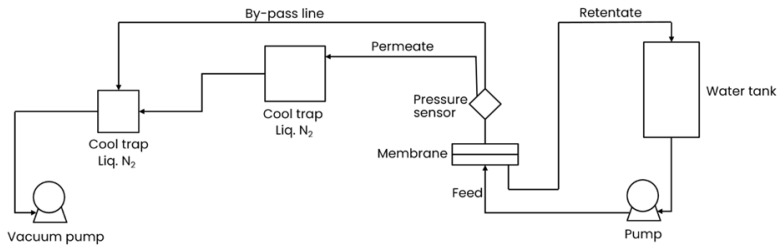
Schematic flow chart of pervaporation.

Pervaporation is a separation technique which utilizes the semi-permeable membrane for preferable selective transport of penetrants that are in the liquid phase on the feed side and the gas phase (vapor) on the permeate side, meaning that liquid evaporation occurs on the immediate interphase of the liquid and feed side of the membrane. The driving force for the vapor transport arises from a chemical potential gradient between feed and permeate sides of membranes created by the application of a vacuum or sweep gas to the permeate side of the membrane causing efficient vapor withdrawal [28]. In this study, pervaporation experiments were performed in addition to the “pressure increase” experiment to provide a comprehensive analysis of membrane performance under different operational conditions with the aim of covering a wide range of feed vapor activity, starting from 100% activity for the case of pervaporation and going from ca. 90 to ca 20% activity in the case of the “pressure increase” experiment. Pervaporation, involving a phase change from liquid to vapor across the membrane, offers distinct insights into the membrane’s efficiency where liquid water is present on the feed side. It is important to note that one of the highlights of this study is the analysis of two water phases, vapor and liquid, that contact the film surface and how this interaction influences vapor transport. Figure 2 shows the schematic flow chart of the pervaporation facility used in this study. A circulation pump provides liquid water circulation from the water tank through the measurement cell containing the membrane under study and back to the water tank. On the permeate side of the measurement cell, a pressure sensor is installed to monitor permeate pressure during the experiment, while the permeate stream is directed into a cold trap cooled with liquid nitrogen for water condensation. To protect the vacuum pump from moisture during the changing of the permeate cold trap, a second cold trap is included in a bypass line. During each measurement session for each membrane sample, 10 measurement points were recorded, with permeate water collected into a cold trap for 5 min at each point. Approximately 15 min were spent between consequent measurements: 5 min for the permeate collection and an additional 10 min to prepare the system for the next measurement, including exchange, evacuation, and accommodation of the new trap to liquid nitrogen temperature. This timing of the experimental sequence is crucial as it was thought to provide an indication of membrane properties’ changes during the experiments. In total, it takes ca. two hours to complete the pervaporation experiment for one membrane. It was assumed that the feed side vapor pressure was 70 mbar, taken as a saturated vapor state at 40 °C, and permeate vapor pressure was considered zero since the vacuum was provided by a rotary pump and additionally by the liquid nitrogen temperature of the cold permeate trap.

## 3. Result and Discussion

### 3.1. Membrane Formation

The aim of this study was to develop a defect-free TFC membranes by coating a thin dense layer on a porous substrate using anthracene maleimide-modified PIM homopolymers with different side groups initially synthesized and studied as thick isotropic films [20]. We successfully prepared selective TFC membranes using the same coating procedure for each homopolymer.

Typically, any defect in the membrane leading to unintended gas flow through pores would result in a noticeable decrease in the ideal selectivity. However, there was no such decrease in selectivity in the experiments based on the example of CO_2_/CH_4_ selectivity as shown in Table S1. It was considered important to keep all parameters consistent during the experiments to make sure the results are reliable and comparable. In this study, attention was given to forming TFC membranes in the same conditions and with the same post-formation treatment, using the same equipment and using the same experimental conditions for all the tests. This careful approach helped avoid errors that could skew the results, ensuring that any observed differences are due to the variables being tested rather than inconsistencies in the setup. This makes the findings accurate and reproducible, providing a solid basis for evaluating the performance and properties of membranes.

### 3.2. Transport Properties of TFC Membranes

#### 3.2.1. Gas and Water Vapor Permeances by “Pressure Increase” Method

In the Results and Discussion section of our study, we provide a detailed analysis of the permeance decay observed for penetrants—water vapor (H_2_O), carbon dioxide (CO_2_), hydrogen (H_2_), and methane (CH_4_)—for thin-film composite membranes studied over a period of at least 20 h. The selection of penetrants for this study was based on specific criteria: H_2_O transport was the primary focus, while H_2_, CO_2_, and CH_4_ were chosen for their wide range of kinetic diameters and differing interactions with the polymer. Analyzing these variations helps us understand the aging processes in the polymer’s selective layer. For all studied membranes, the order in which penetrants were applied to the membrane sample was the same: CH_4_, H_2_, CO_2_, H_2_O. Water, even being close to the saturation state, was considered as penetrant and not able to change the state of the polymer of the selective layer due to, e.g., the formation of molecule clusters within the free-volume elements and, thus, no investigation on this matter was carried out [29,30]. The measurement method involved exposing the membranes to a high vacuum, provided by the turbomolecular pumping unit, for full penetrant desorption during the multiple gas change procedures, which did not occur during the pervaporation experiment and can be a trigger for the fast aging process in the selective layer. As mentioned above, each TFC membrane sample was continuously studied for gas and water vapor transport properties during the period of at least 20 h with multiple full evacuations and exposures to different penetrants and, after this, the sample was removed from the experimental facility. Once removed from the experimental setup, the membrane sample could not be reused due to potential damage to the selective layer. This damage could occur from detaching the layer from the UF support or removing the O-ring from the membrane surface.

In our initial study [20], we observed that the introduction of maleimide derivatives with various side chain groups significantly influenced water vapor transport through the studied polymers. This effect was primarily due to the polymer polarity change caused by the imide groups, which altered facilitated hydrogen bonding with water molecules’ interaction with polymers. These observations were made for the case of dense thick films of homopolymers, where it was noted that the polymers with the “-methyl” side group exhibited the highest water permeability. The “-methyl” side group, having the shortest alkyl side chain, impacted the polymer polarity the most among those tested. The increase in the number of carbon atoms in linear alkyl side groups was found to decrease water permeability.

When compared with examples where linear alkyl groups were replaced with bulkier groups such as “-*i*-propyl” or “-*t*-butyl”, it was observed that bulky, in comparison to “-methyl”, side groups influenced both diffusivity and solubility and, hence, gas permeability. The introduction of bulky groups significantly increased the free volume, which consequently increased the diffusion coefficient.

**Figure 3:**
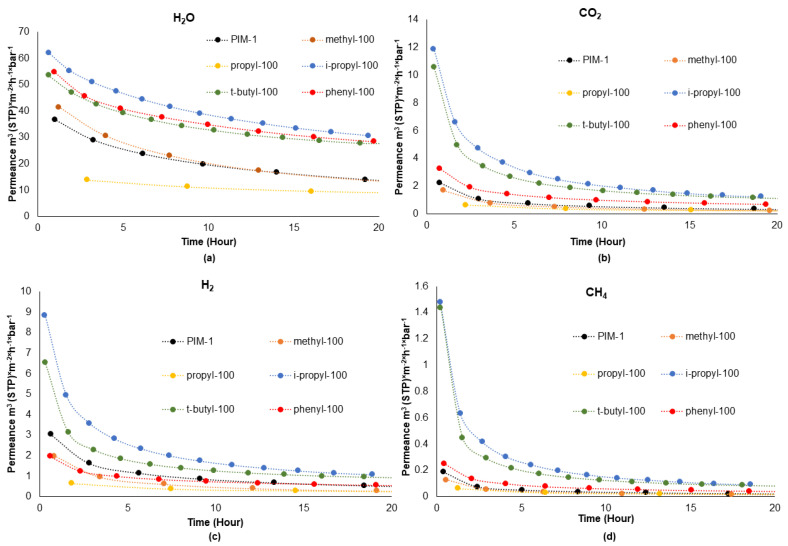
Gas and water vapor permeances of TFC membranes over 20 h. (**a**) water permeance, (**b**) carbon dioxide permeance, (**c**) hydrogen permeance, (**d**) methane permeance.

In this study, the experimental results obtained for TFC membranes with selective layers of homopolymers show differences from those obtained for thick isotropic films. Figure 3 shows the gas permeance decay of each homopolymer-based TFC membrane observed over the span of 20 h. The complete measurements for each TFC membrane can be found in Figure S1.

Among the studied TFC membranes, those with selective layers of *i*-propyl-100 and *t*-butyl-100 homopolymers exhibited the highest gas permeances. Based on their kinetic diameter, CO_2_ and CH_4_ are larger molecules while H_2_ and H_2_O are smaller in size (H_2_O: 2.65 Å; H_2_: 2.89 Å; CO_2_: 3.3 Å; CH_4_: 3.8 Å) [31]. If permeance is evaluated according to molecule size, we see that the most permeant homopolymers for CO_2_ and CH_4_ are *i*-propyl-100 and *t*-butyl-100. This outcome is attributed to the increased free volume created by the incorporation of bulky groups, which in turn enhances the permeance of larger molecules. Previous studies have demonstrated that incorporating bulky groups into polymers enhances their free volume [32,33]. This increase in free volume improves diffusivity, which in turn indirectly boosts gas permeability, as it is a function of diffusivity. Notably, *i*-propyl-100 and *t*-butyl-100 displayed an extremely rapid decline in permeance, which was particularly pronounced within the first two hours of observation. This sharp decrease is likely attributed to the formation of large free-volume elements due to the presence of the bulky side groups in these homopolymers and the impact of the high vacuum applied to the membrane sample during the “pressure increase” causing full degassing of the selective layer and thus enabling easier rearrangement of the free volume within these polymers. Swaidan et al. reported that polymers with bulky and rigid groups are more significantly affected by physical aging. Additionally, it was observed that H_2_, with its smaller kinetic size, experiences a smaller reduction in permeance due to physical aging compared to an oxygen molecule which is larger in kinetic diameter [34]. These observations are critical for understanding the behavior of TFC membranes in different applications, particularly under conditions where rapid changes in environmental conditions or gas exposures occur.

When examining the permeance of water vapor, which is the smallest molecule among the studied penetrants, one observes that phenyl-100 is the most permeable homopolymer after i-propyl-100. Due to its chemical nature, water can be considered differently in the permeance mechanism compared to other gasses. Water molecules can form hydrogen bonds with each other, creating unique scenarios during gas transport. These include the formation of clusters of water molecules and plasticization due to the interaction between water molecules and the polymer matrix. Cluster formation is prevalent in hydrophobic environments where water–polymer interaction is weak, whereas plasticization is observed when water–polymer interaction is significant [30,35]. A detailed discussion of water clustering is in the following sections of this paper. Although the homopolymers used in this study are hydrophobic due to their backbone, the phenyl side group in the phenyl-100 homopolymer enhances its affinity for water, as demonstrated by the exceptionally high solubility coefficient of H_2_O in this polymer in comparison to that of other substituted homopolymers. The interaction between the aromatic ring functional group in the phenyl-100 and water is likely enhanced by possible OH-π electron interactions, as suggested in other studies [36,37]. This interaction can be expected to increase the affinity of phenyl-100 toward water. Therefore, in addition to the prominent free-volume effect observed in *t*-butyl-100 and *i*-propyl-100, the water vapor permeance of phenyl-100 is evaluated from a distinct perspective.

The variation in polymer order between gas permeances of TFC membranes and permeability coefficients of thick films used in our previous study [20] could arise from the method of TFC membrane sample preparation. Thick films of polymers were prepared by as-slow-as-possible evaporation of the solvent from the polymer solution, while TFC membrane preparation involved rapid evaporation of the organic solvent from the polymer solution layer deposited onto the porous UF membrane. The later method involves significant changes in the polymer solution temperature, while the polymer concentration changes from 1% to 100% by weight in just a few seconds due to the loss of solvent to the ambient; such drastic changes have been previously reported [38].The change in solution temperature due to intensive evaporation and possible differences in polymer solubility in the organic solvent caused by the presence of different side groups in the polymer can be reasons for the induced difference in polymer packing when a selective layer of TFC membrane is formed.

Regarding permeance decay over time, water exhibited a much slower permeance decay rate compared to the other gasses. This slower decay suggests that either water interacts differently with the polymer matrix or its transport mechanism through the membrane is distinct from that of the other tested gasses. In contrast, CO_2_, H_2_, and CH_4_ showed comparable rates of permeance decay. Accordingly, for the PIM-1 TFC membrane, the permeance loss for CO_2_ and CH_4_ within 20 h was about 7 and that for H_2_ was about 5.9, while that for H_2_O was only 3.9.

**Figure 4:**
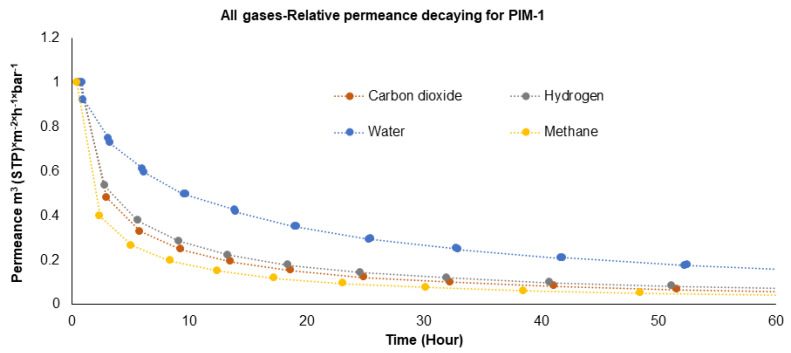
Decay of relative gas and water vapor permeance of PIM-1 TFC membrane.

To further investigate the comparison of water vapor permeance with other gasses, an analysis of relative gas permeance reduction can be made by focusing on PIM-1. Figure 4 shows the decay of the relative permeances of four penetrants measured with the PIM-1 TFC membrane over 60 h. For all gasses, the most drastic permeance decrease occurs in the first 10 h. CH_4_, the largest molecule, was the most affected by selective layer aging. It showed a permeance drop of up to 60% in the first two hours of measurement. This was followed by CO_2_ and H_2_, but H_2_O showed a permeance drop of 30% in the first two hours compared to the other gasses. It has been demonstrated in previous studies that thin films are affected by physical aging much more rapidly than dense films [39,40]. In examining the relative permeance decay of gasses through the PIM-1 polymer, as shown in the graph, several key points emerge regarding the interaction between these gasses and the polymer and how these interactions affect their aging and transport properties over time.

As can be seen in the graph, water vapor exhibits a notably slower aging process compared to other gasses. This slower decay can be attributed to the highly associated nature of water molecules, which tend to form hydrogen bonds with each other. These bonds likely facilitate a more “organized” movement of water molecules through the selective layer membrane, akin to the anomalous transport behavior observed in carbon nanotubes (CNTs) [41,42], where water moves as a coordinated chain or belt. This property of water significantly impacts its permeance stability over time and can be the only explanation why H_2_O molecules do not “feel” changes in the free volume. H_2_, which has a kinetic diameter very similar to that of H_2_O, shows much higher permeance decay than H_2_O, followed by CO_2_ and, finally, CH_4_. These three gasses do not form bonds with the polymer or among themselves, which could contribute to a faster decay in their permeance as they are less able to maintain structured paths through the polymer. Moreover, CO_2_, being the most soluble of the gasses tested, demonstrates a decay pattern that, while faster than water, is slower than hydrogen and methane. The solubility of CO_2_ in the polymer likely contributes to its relatively slower decay, as the gas can be absorbed more readily into the polymer matrix, thereby mitigating rapid permeance loss.

Consequently, it is important to bear in mind that the physical properties of the gasses, such as solubility and the ability to form associative interactions, play critical roles in determining how quickly the permeance decays over time through a specific polymer matrix like PIM-1. These insights are crucial for applications where the long-term stability of gas separation and transport properties are of utmost importance.

#### 3.2.2. Vapor Activity

**Figure 5:**
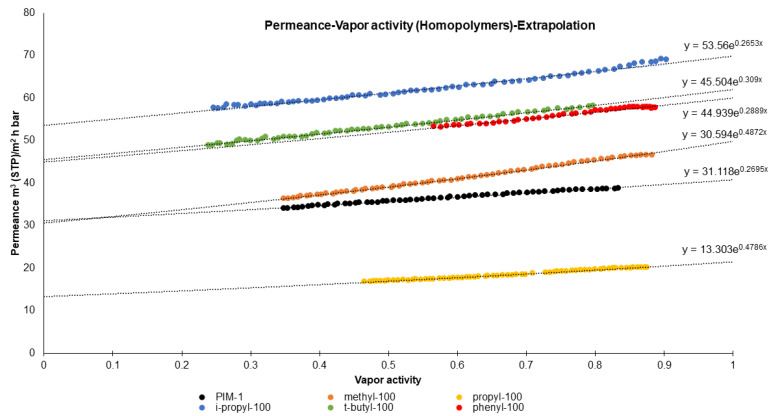
Vapor permeance as function of vapor activity for TFC membranes.

Another aspect that can be examined is water vapor permeance as a function of feed vapor activity. Figure 5 renders a series of plots for different homopolymers and demonstrates that the permeance of all homopolymers, including PIM-1, increases with vapor activity, which is expected as higher vapor activity generally increases the driving force for permeation. Davis et al. reported that at higher vapor activities, water molecule association increased, followed by an enhancement in water permeability [29]. Unfortunately, design limitations of the experimental facility prevent experiments at vapor activity above 90%. But the acquired data demonstrate a clear upward trend in permeance as vapor activity increases. The trend can be fitted by the exponential function with the coefficient of determination above 98%. The use of the exponential function is justified by the consideration of vapor as penetrant due to being close to the condensation, and the equation of the “Free Volume Model” can be applied instead of apparent linear regression [43]. Taking into account no significant swelling of the selective layer by water vapor, clearly indicated by small values of the exponent coefficient, and the fact that the experimental data start at 90% of vapor activity, one can assume the validity of the applied function to be extended up to 100% activity and thus to prognose vapor permeance at saturation conditions.

#### 3.2.3. Water Permeance Measurement by Pervaporation Method

In this part, the results of pervaporation experiment will be discussed as the second method employed to investigate water vapor transport in TFC membranes. The results of the pervaporation experiment are essential to understand vapor transport through polymeric membranes at conditions of 100% relative humidity at the feed side interphase and to compare results to that obtained during the “pressure increase” experiment.

Table 1 shows the results of the pervaporation experiment for each TFC membrane. Values for each membrane are the average of 10 separate data points, collected during continuous membrane exposure to liquid H_2_O over 2.5 h. The water penetrated through the membrane was collected in the cold trap, the trap was weighted on the balance and the average mass value of penetrant collected over 5 min is presented as “Water flux (g)”. In order to calculate water vapor permeance in the same dimension as in the “pressure increase” experiment, the mass was converted to the volume of vapor in STP conditions; the driving force was assumed as 70 mbar on the feed side and zero pressure on the permeate side. The calculated value is presented as “pervaporation permeance” and can be compared to the “Pressure increase permeance”, the values of which were approximated to the 100% vapor activity using fitting equations from Figure 5.

The pervaporation results showed much higher values than those of the “pressure increase” measurements. The substantial difference in results prompted further exploration of the processes occurring within the membrane systems.

The discrepancy of possessing higher water flux in pervaporation than “pressure increase” measurement could be attributed to the abovementioned clustering of water molecules within the free volume of the polymers under investigation, which plays a crucial role. It is most likely that a scenario similar to the transport of water molecules in CNTs [41,42,44] is occurring in our polymer system where we operate the membranes in pervaporation. This particular transport resulting from the formation of water clusters can be explained by the fact that water molecules, owing to their small size and ability to form hydrogen bonds, exhibit a unique “dragging” effect that allows them to pass through bottlenecks between large free-volume elements without having to navigate independently through the polymer cavities [45].

In our pervaporation setup, the initial conditions are such that water molecules are already at saturation point when they encounter the free-volume elements, which differs significantly from conditions in “pressure increase” experiments. This saturation provides conditions for the formation of water molecules aggregates already on the feed/membrane interphase without the need to change the state of molecules and continues within the free-volume elements, a behavior that has been observed in studies of alcohol molecules in perfluorinated polymers, which are known for their extreme hydrophobicity [46]. It can therefore be assumed that the clustering of water molecules plays a substantial role in the transport properties observed in TFC membranes under different experimental conditions. The scope of this study was limited in terms of the availability of quantitative analysis or modeling since our current analysis remains qualitative. Further research focusing on the molecular dynamics might offer valuable insights into designing and optimizing membrane materials for specific applications where water transport efficiency is crucial. This could provide a more comprehensive understanding of the mechanisms driving permeance in thin-film composite membranes.

**Figure 6:**
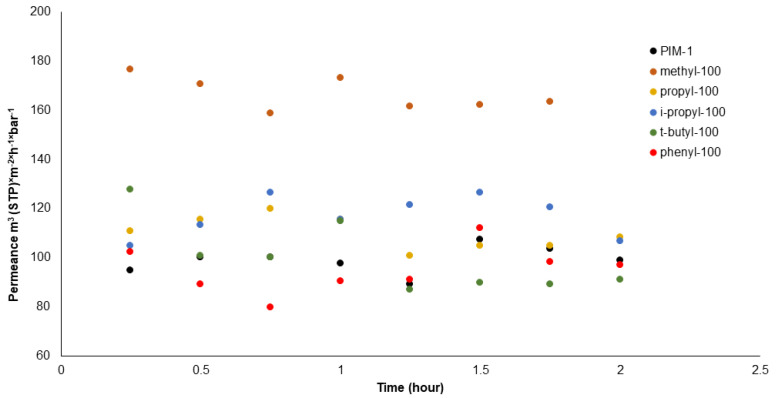
Water flux values from pervaporation over 2.5 h.

To understand how the membranes react to aging under two different measurement methods, it would be beneficial to analyze the time-dependent changes observed in the pervaporation experiment results. Figure 6 exhibits the pervaporation measurement results over a span of 2.5 h for each membrane. It can be seen that the curves display a slight scattering trend in water permeance across the membranes. While the trend does show minor fluctuations as in the case of methyl-100, the permeance decay is not as pronounced as that observed in “pressure increase” experiments.

This steady water permeance over time can be attributed to the experimental method of pervaporation where the feed side of the membrane is constantly exposed to liquid water, maintaining an interface between water and the polymer at 100% humidity during the pervaporation process. Such conditions are conducive to the condensation or association of water molecules and, thus, the formation of water clusters already on the feed interphase of the selective layer of the membrane. Consequently, it can be inferred that the presence of these clustered or associated water molecules within the selective layer likely plays a critical role in moderating the decay of the free-volume elements of the membrane. Basically, the void within the polymer matrix is occupied by these clusters, which hinders the rapid decay typically seen in “pressure increase” measurements of hydrogen or carbon dioxide. This effect of water molecule clusters on the stability of the polymer structure supports the concept that the clustered state of water helps preserve the integrity of the polymer, preventing the free volume from collapsing too quickly, a phenomenon corroborated by findings in other studies such as those referenced by Metz et al. [47]. In summary, the pervaporation data suggest that direct exposure to liquid water and the resulting high humidity at the membrane interface play crucial roles in sustaining the polymer structure against rapid degradation, primarily through the stabilizing influence of water molecule clustering within the polymer matrix.

**Figure 7:**
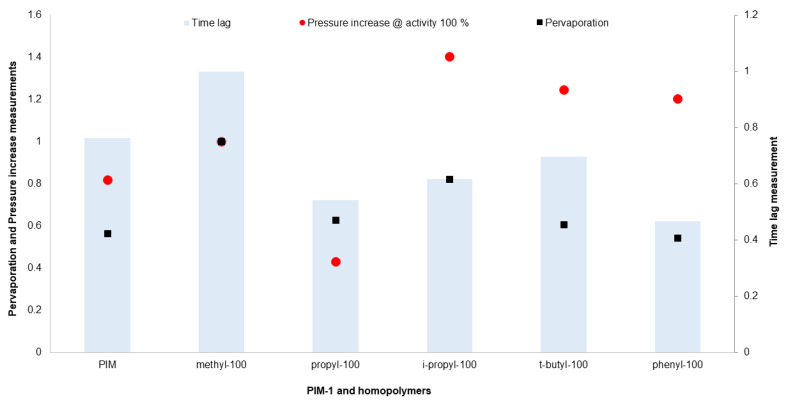
Comparison of relative permeance data of pervaporation, “pressure increase” and relative permeability of “time lag” measurement.

Up to this point in the current study, the water vapor transport of TFC membranes prepared with previously synthesized polymers has been investigated and evaluated in two different applications. In order to extend the scope of this comparison, it would be useful to compare the findings with the “time lag” results from the previous study [20]. With this aim, Figure 7 presents a comparative analysis of three distinct measurement methods: pervaporation, “pressure increase”, and “time lag”. Each data point represents relative values to the performance of methyl-100-based films and TFC membranes. It is crucial to understand that the values indicated are not directly comparable across different methods. For instance, in pervaporation, each membrane value was divided by methyl-100 within its own measurement group. Therefore, all three measurements have the same data on methyl-100 as being equal to 1. The data include relative “pressure increase” values extrapolated to 100% vapor activity to facilitate comparison with pervaporation results. This visualization aims to show the relative changes in properties of different polymers within each method by normalizing the results against the performance of the methyl-100.

Observations indicate that while the trends in “time lag” and pervaporation measurements appear similar, vapor permeation by pressure increase displays notable differences. This discrepancy can most likely be attributed to the state of the polymer, which varies significantly between the methods. In time lag measurements, the thick polymer film is as relaxed as possible, allowing for slow evaporation and generally slower aging, which is a phenomenon strongly influenced by film thickness, as discussed in studies such as those by McCaig et al. [48,49]. On the contrary, in TFC membranes which are used in pressure increase measurements, the rapid drying of the polymer solution leads to a thermodynamically unstable state on the surface of the membrane. This instability is compounded by the stress caused by the interface of the polymer, potentially accelerating the degradation of free volume. However, this might be seen as a rearrangement of the polymer structure rather than a mere loss of free volume. Moreover, the influence of the interface to air between the polymer and gas in thick films is minimized compared to that in TFC membranes. According to the observations of this study, it can be assumed that in TFC and thick films, the top layer of the polymer might age similarly, but this does not significantly affect overall performance in thick films. Fundamentally, this graph not only highlights the variations in polymer behavior across different measurement techniques but also underlines the complex interplay of polymer structures, environmental conditions, and measurement methodology in determining polymer performance.

## 4. Conclusions

This study describes the comparison of gas and water vapor permeances of PIM-derivative TFC membranes obtained under different operating conditions. The different operating conditions include the pervaporation and gas permeation test system “pressure increase” methods. In addition to this, the comparison was also evaluated from the perspective of film thickness by adding “time lag” measurements of thick films obtained from our previous study. Accordingly, TFC membranes were prepared with PIM-1 and homopolymers with different side groups. With these TFC membranes, CO_2_, H_2_, CH_4_ and water vapor permeances were measured with the “pressure increase” measurement. The permeance values were evaluated over a period of 20 h and the aging of the TFC membranes was investigated. In addition to this measurement, the TFC membranes were subjected to pervaporation experiments, water flux was measured and the results were discussed in detail. By comparing the aging behavior and separation performance of thin-film composite membranes against thick films, our work lays the groundwork for future research aimed at optimizing these advanced materials for sustainable and efficient separation processes. The results from the pervaporation experiments are particularly promising, showing much higher water vapor transport rates compared to those observed in pressure increase setups. This suggests that pervaporation could be a viable alternative to traditional porous membrane applications in distillation, especially considering the high permeance values achieved. These findings support the feasibility of using non-porous membranes in applications where high efficiency in water vapor transport is required. However, a critical aspect of implementing such systems involves understanding long-term membrane stability under pervaporation conditions. The current study indicates that while pervaporation offers higher transport rates, the longevity and durability of membranes under continuous operation remain uncertain due to the intensive nature of the required experiments. Such experiments necessitate continuous supervision, primarily because of the need for constant provision of liquid nitrogen, making them labor-intensive and challenging to conduct over extended periods. To fully realize the potential of pervaporation and other non-porous membrane techniques in industrial applications, further research is necessary to assess long-term stability and performance. This would involve designing experiments that can operate safely and effectively over long durations to monitor aging processes and ensuring that the membranes can withstand prolonged operational conditions without significant degradation. In conclusion, while the initial findings are encouraging, confirming the practical viability of non-porous membranes for distillation and similar applications will require more extensive and sustained experimental work. This future research will be crucial in moving from theoretical and initial experimental successes to reliable, scalable industrial applications.

## Figures and Tables

**Table 1:** Absolute values of converted water flux obtained by pervaporation.

TFC Membranes	Water Flux (g)	Pervaporation Permeance m^3^(STP)/m^2^ × h × bar	“Pressure Increase” Permeance m^3^(STP)/m^2^ × h × bar
PIM-1	1.65	98.8	40.7
methyl-100	2.92	174.7	49.8
propyl-100	1.84	109.9	21.5
*i*-propyl-100	2.40	143.5	69.8
*t*-butyl-100	1.77	105.9	61.8
phenyl-100	1.98	94.9	50.0

## Data Availability

Data are contained within the article and Supplementary Materials.

## Supplementary Materials

The following supporting information can be downloaded at https://www.mdpi.com/article/10.3390/polym16202932/s1. Table S1: Gas and water vapor permeance of PIM-1 at different hours, Figure S1: Gas and vapor permeance decay of homopolymers—complete measurement.
