# Supplementary material for: Comparative Study of Polymer of Intrinsic Microporosity-Derivative Polymers in Pervaporation and Water Vapor Permeance Applications
Source: Polymers (Basel). 2024 Oct 18;16(20):2932. doi: 10.3390/polym16202932 (PMC11511032; doi:10.3390/polym16202932)
Supplement: Supplementary file 1 [file polymers-16-02932-s001.zip › polymers-3238547-supplementary.pdf]

polymers MDPI

Supplementary Information

Comparative study of PIM derivative polymers in pervaporation and water vapor permeance applications

Esra Caliskan[1], Sergey Shishatskiy[1], Volkan Filiz[1,] *

1 Helmholtz-Zentrum Hereon, Institute of Membrane Research, Max-Planck-Str. 1, 21502 Geesthacht, Germany

* Correspondence: volkan.filiz@hereon.de; Tel.: +49-41-5287-2425

Table S1. Gas and water vapor permeance of PIM-1 at different hours

Gas permeance (m^3(STP) m^{-2} h^{-1} bar^{-1})		
	@hour=1	@hour=20
CH_4	0.128	0.018
CO_2	2.16	0.306
H_2	2.830	0.482
H_2O	47.8	12.3

Gas selectivity		
	@hour=1	@hour=20
H_2/CH_4	22.2	26.7
H_2/CO_2	1.31	1.57
CO_2/CH_4	16.9	17.0
H_2O/CH_4	373.4	683.3
H_2O/CO_2	22.1	40.2
H_2O/ H_2	16.9	25.5

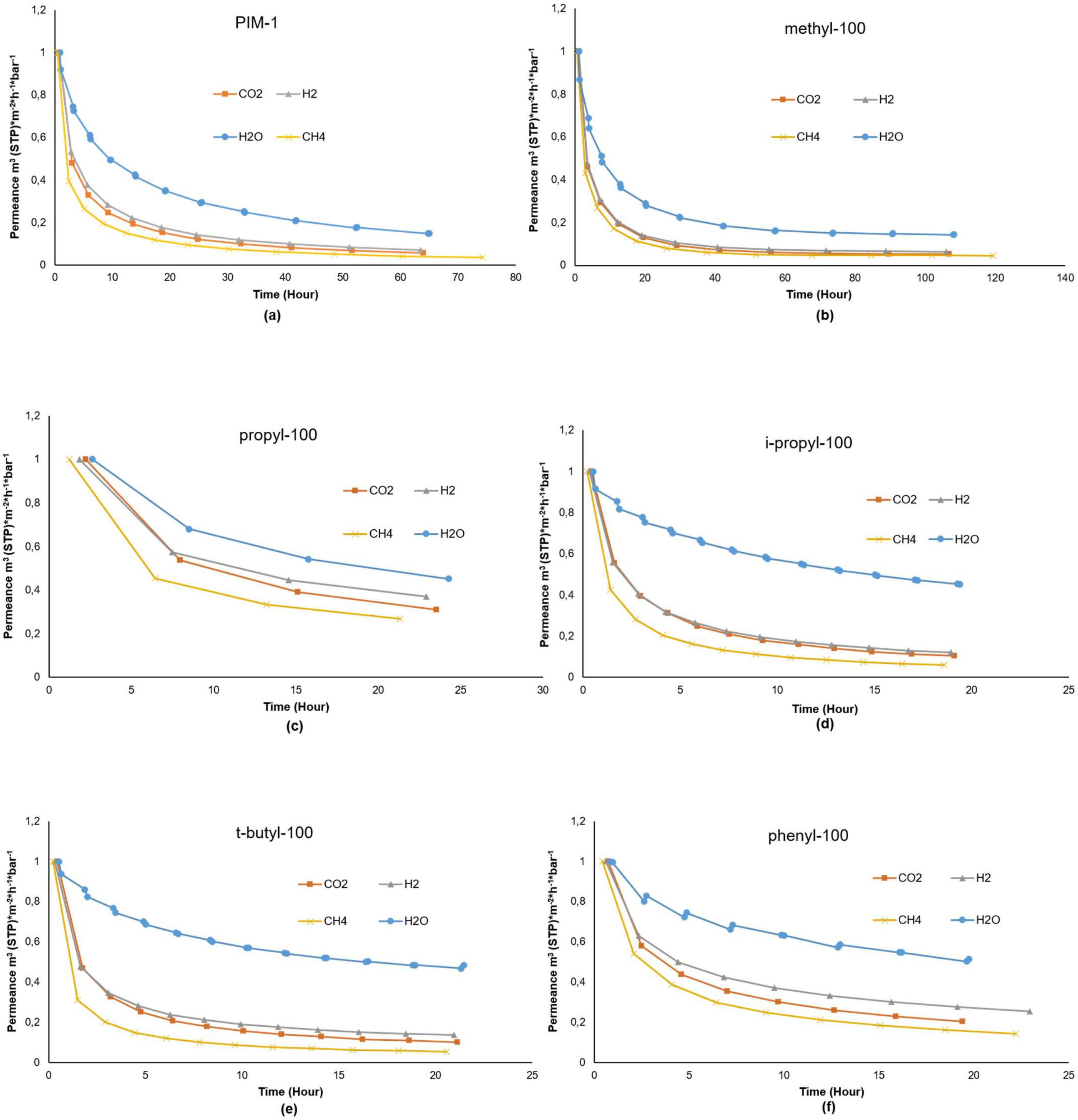

Figure S1: Gas and vapor permeance decay homopolymers-complete measurement
